# Synchronization of rumen degradable protein with non-fiber carbohydrate on microbial protein synthesis and dairy ration digestibility

**DOI:** 10.14202/vetworld.2022.252-261

**Published:** 2022-02-05

**Authors:** Annisa Rosmalia, Idat Galih Permana, Despal Despal

**Affiliations:** 1 Nutrition and Feed Science Study Program, Faculty of Animal Science, IPB University, Bogor 16680, West Java, Indonesia; 2 Department of Animal Nutrition and Feed Technology, Faculty of Animal Science, IPB University, Bogor 16680, West Java, Indonesia

**Keywords:** dairy ration digestibility, microbial protein synthesis, rumen degradable protein, rumen undegradable protein, synchronization

## Abstract

**Background and Aim:**

Dairy ration formulations should consider the synchronization of the rumen degradable protein (RDP) to rumen undegradable protein (RUP) ratio (RDPR) with non-fiber carbohydrate (NFC) to achieve optimum microbial protein synthesis (MPS), reduce feed costs, and reduce N excretion to the environment. This study aimed to investigate the effect of RDPR and NFC synchronization on *in vitro* digestibility, fermentability, and MPS.

**Materials and Methods:**

The experiment used a 3×3 factorial randomized block design with four replications. The first factor was RDPR (RDPR1=50:50; RDPR2=55:45; RDPR3=60:40) and the second factor was NFC levels (NFC1=30%, NFC2=35%, NFC3=40%). The experimental diets were evaluated using a two-stage *in vitro* method. The examined parameters included rumen pH, NH_3_ concentration, total volatile fatty acid (VFA) concentration, the molar proportion of VFAs, rumen microbes (protozoa and total bacteria population), and MPS. Data were analyzed using ANOVA, followed by the Duncan test.

**Results:**

The results show that neither RDPR nor NFC affected rumen pH, NH_3_, total VFA, and the rumen microbe population. The interaction between RDPR and NFC affected the molar proportion of acetate, iso-butyrate, and n-valerate. The combination of RDPR1 and NFC1 produced a lower molar proportion of acetate (49.73%) than the other treatment combinations (>54%). The acetate to propionate ratio was influenced by the NFC levels, in which NFC2 and NFC3 produced the highest ratio (p<0.05). MPS was affected by RDPR and NFC, but not by their interaction. Treatments NFC2 and RDPR3 produced the highest MPS. NFC affected the dry matter and organic matter digestibility (DMD and OMD), with treatment NFC3 resulting in the highest DMD and OMD.

**Conclusion:**

The combination of a 60:40 RDPR with 35% NFC resulted in the best synchronization of protein and energy available for MPS and digestion activity in the rumen.

## Introduction

Ruminant protein requirements can be divided into rumen degradable protein (RDP) and rumen undegradable protein (RUP). RDP is required for microbial protein synthesis (MPS) as a source of nitrogen (N) which plays a role in supplying protein to dairy cattle [1]. In addition to protein and nutrient sufficiency, dairy ration formulations should consider the RDP to RUP ratio (RDPR) for optimal MPS. Rumen microbes need the RDPR as a source of N and energy as a source of carbon (C); both N and C should be available in the right proportion to improve MPS efficiency [2,3]. Therefore, degradable protein and fermentable carbohydrates should be provided. Non-fiber carbohydrate (NFC) provides readily available carbohydrate (fermentable) that contains sugars, starch, fructans, pectin substance, galactans, and β-glucans [4]. NFC is calculated using the following formula: NFC=100– neutral detergent fiber – crude protein – ether extract – ash. The difference between NFC and non-structural carbohydrate is the neutral detergent soluble fiber (pectin, galactans, and β-glucans), which is included in NFC [4,5]. NFC can be provided in rations at 30-45% of dry matter (DM) [6].

The synchronization between RDPR and NFC is closely related to ruminal fermentation, which requires an optimum level. The optimum RDPR and energy levels are beneficial to milk protein production due to the increase of MPS [7]. The previous studies have reported that the NFC source and RDP synchronization affect rumen fermentation, microbial growth, lactation performance, and blood parameters in dairy cows [6,8]. High NFC diets synchronized with RDP reduce N losses [9]. On the other hand, unsynchronized RDPR and NFC lead to feed inefficiency, high feed costs, and high excess N pollution to the environment [10]. The National Research Council (NRC) [11] recommends that the optimal RDPR for dairy cows is 60:40. A previous study reported that feeding dairy cows with RDPR of 65:35 led to better milk production than RDPR of 60:40 [12].

Conversely, dairy rations with RDPR below the NRC recommendation [11] decrease milk yield, milk fat, and milk protein due to insufficient RDP available for rumen microbial growth [13]. Low RDPR depresses DM intake and milk production [14]. Moreover, RDPR of 63:37 is also more efficient in early lactating dairy cows, providing optimal protein for milk production and minimum urinary N excretion [15]. Dairy rations containing high NFC increase ammonia N utilization and MPS [16]. In addition, supplying energy as high NFC improves protein metabolism in the body, impacting milk production and quality, especially milk protein [17]. Feed efficiency and N conversion are also improved with a high NFC diet [18].

Information concerning the RDPR and NFC in dairy feedstuffs has mainly been investigated in developed countries, which use different feedstuffs than the tropical dairy ration [19-21]. Moreover, synchronization index assessments conducted in a temperate region may not be suitable for application to tropical conditions. Thus, the objective of this study was to evaluate the effects of tropical dairy rations based on RDPR and NFC synchronization on digestibility, fermentability, and MPS using an *in vitro* method.

## Materials and Methods

### Ethical approval

The cannulation surgery of animal was carried out by a licensed veterinarian and followed the protocol handling and care of animal according to the IPB University Animal Ethics Committee.

### Study period and location

This study was conducted from January to April 2021 at the Laboratory of Dairy Nutrition, Faculty of Animal Science, IPB University.

### Experimental diet

The experiment used a 3×3 factorial randomized block design; the first factor was RDPR (RDPR1=50:50; RDPR2=55:45; RDPR3=60:40) and the second factor was NFC levels (NFC1=30%; NFC2=35%; NFC3=40%). The rations contained 40% forage and 60% concentrate (DM basis). The experimental dairy rations were formulated based on the requirements and energy-protein balances from the NRC [11]. The RDPR of the ration was calculated using a local database generated from a previous study in our laboratory, whereas NFC was calculated according to the methodology of Mertens [5]. The feed ingredients and chemical composition of the rations are shown in Table-1 [22].

**Table 1:** The feed ingredients and chemical composition of dairy rations^1^.

Items	NFC1			NFC2			NFC3		
		
	RDPR1	RDPR2	RDPR3	RDPR1	RDPR2	RDPR3	RDPR1	RDPR2	RDPR3
Ingredients, % of diet DM									
Napier grass	40.00	40.00	40.00	40.00	40.00	40.00	40.00	40.00	40.00
Corn	11.55	10.50	10.10	10.00	10.00	10.00	11.85	11.90	11.40
Rice bran	6.80	9.00	8.00	4.39	4.20	3.50	0.00	0.00	0.00
Pollard	6.65	6.20	7.25	1.50	1.10	1.50	0.00	0.00	1.00
Cassava meal	11.50	11.50	11.95	22.00	22.00	22.25	32.60	31.60	31.40
Soybean meal	1.00	5.40	10.60	2.36	8.75	13.25	1.00	8.00	12.20
Corn gluten meal	10.00	5.00	0.20	9.80	4.00	0.20	12.00	6.00	2.00
Copra meal	3.00	6.00	7.70	4.00	4.00	4.30	0.00	0.50	0.00
Coffee husk	7.50	4.40	2.20	3.95	3.95	3.00	0.55	0.00	0.00
Calcium carbonate	1.00	1.00	1.00	1.00	1.00	1.00	1.00	1.00	1.00
Dicalcium phosphate	0.50	0.50	0.50	0.50	0.50	0.50	0.50	0.50	0.50
NaCl	0.50	0.50	0.50	0.50	0.50	0.50	0.50	0.50	0.50
Chemical composition									
DM, %	92.43	92.70	92.47	92.19	91.85	90.63	90.04	90.18	90.37
Ash, % DM	9.08	9.84	9.87	8.86	9.43	9.80	7.38	7.65	8.08
EE, % DM	1.66	1.72	2.18	1.88	1.65	1.64	0.88	0.80	0.87
CP, % DM	15.83	16.10	15.40	16.43	15.98	15.97	16.42	15.89	15.48
CF, % DM	17.43	16.59	15.96	15.80	15.90	15.74	13.72	14.71	13.50
NFE, % DM	56.00	55.74	56.58	57.03	57.03	56.84	61.60	60.95	62.07
TDN^2^, %	64.42	64.88	65.73	66.83	65.88	65.73	69.39	67.81	68.71

1 Experimental dairy rations: NFC1=NFC 30%; NFC2=NFC 35%; NFC3=NFC 40%; RDPR1=50:50 RDP: RUP ratio; RDPR2=55:45 RDP: RUP ratio; RDPR3=60:40 RDP: RUP ratio. DM=Dry matter, EE=Ether extract; CP=Crude protein, CF=Crude fiber, NFE=Nitrogen free extract, TDN=Total digestible nutrient.

2 TDN=2.79+(1.17×%CP)+(1.74×%EE)-(0.295×%CF)+(0.81×%NFE) [22]

### *In vitro* procedure and sample measurement

This study used the two-stage method procedure of Tilley and Terry [23]. Rumen liquor, as an inoculant source, was collected from ruminal fistulated Frisian Holstein (BW±510 kg) before morning feeding. The rumen liquor was filtered using two layers of cheesecloth. A 0.5 g sample was placed in a fermenter tube, to which 40 mL pre-warmed McDougall buffer solution and 10 mL rumen liquor was added. The tube was aerated with CO_2_ to generate anaerobic conditions, capped with a ventilated rubber stopper, and placed in a shaker water bath at 39°C for 48 h.

After 4 h of incubation, 0.5 mL and 1 mL of fermentation liquid were separated for total bacterial and protozoa counts, respectively. Two drops of HgCl_2_ were added to the tube to halt fermentation activity. The tube was centrifuged at 1409 x g for 15 min. The supernatant was collected and frozen, which was later used to determine NH_3_ concentration, total volatile fatty acid (VFA) concentration, and the molar proportion of VFAs.

For digestibility measurements, a similar incubation procedure was conducted. After 48 h of *in vitro* rumen incubation, two drops of HgCl_2_ solution were added to halt the microbial activity. Then, the sample residue obtained after centrifugation was combined with 50 mL of 0.2% pepsin-HCl solution and incubated at 39°C for 48 h. After 48 h of pepsin-HCl incubation, the samples were filtered using a pre-determined weight of Whatman filter paper No. 41 using a vacuum pump. Afterward, the paper was dried to determine the DM residue and incinerated to determine the ash residue.

Rumen pH was measured using a pH meter (Hanna Instruments, HI98191, Romania). The supernatant was thawed at room temperature (25°C) before analyzing NH_3_, total VFAs, and MPS. The NH_3_ concentration was determined using the Conway microdiffusion method, whereas the total VFA concentration was determined using the steam distillation method; both methods were similar to those used by Riestanti *et al*. [24]. Individual VFA (molar proportion) was analyzed using gas chromatography, using a procedure described by Yulistiani *et al*. [25]. Methane production was estimated from the individual VFA using the formula: CH_4_=0.45×acetate–0.275×propionate+0.40×butyrate [26]. After coloring using Trypan blue formal saline, rumen protozoa were counted using a microscope (XSZ-107BN binocular biological microscope, BW Optics, China) with 4×10 magnification. Total rumen bacteria were counted using serial dilution and cultured for 24 h in BHI media (HiMedia, India). The counting followed the Ogimoto and Imai methods as described byHu *et al*. [27] and Wahyudi *et al*. [28].

### Statistical analysis

Data were analyzed by one-way analysis of variance using the statistical analysis system program OnDemand for Academic (SAS Inc., NC, USA). Statistical significance was set at p<0.05. Differences between means were compared usingDuncan’s multiple range test.

## Results

### Fermentation characteristics

The observed fermentation characteristics (rumen pH, NH_3_ concentration, and total VFA concentration) are shown in Table-2. There was no interaction between the two factors (RDPR and NFC) and no significant effects of the main factors on rumen pH, NH_3_, and total VFA concentration (p>0.05). The rumen pH values in this study ranged from 6.92 to 6.97, which was within the normal range (6.0-7.0) according to Poulsen *et al*. [29]. The NH_3_ and VFA concentrations also fell within the normal ranges, with values ranging from 7.60 to 8.11 mM and 100.96 to 121.20 mM, respectively [30,31].

**Table 2:** pH, NH_3_, and total VFA concentration of the experimental diet.

Parameters	RDP	NFC			Average±SD

		NFC1	NFC2	NFC3	
pH	RDPR1	6.98	6.95	6.92	6.95±0.04
	RDPR2	6.93	6.93	6.88	6.91±0.05
	RDRP3	6.93	6.97	6.92	6.94±0.04
	Average±SD	6.95±0.04	6.95±0.05	6.91±0.04	
NH_3_ (mM)	RDPR1	7.86	7.60	7.59	7.68±2.64
	RDPR2	7.79	7.81	8.11	7.90±1.91
	RDRP3	8.09	7.56	8.09	7.92±2.02
	Average±SD	7.91±2.70	7.66±2.02	7.93±1.81	
VFA total (mM)	RDPR1	100.96	121.20	110.71	110.96±20.81
	RDPR2	130.40	114.67	109.02	118.03±22.70
	RDRP3	114.75	121.02	109.23	115.00±19.27
	Average±SD	115±23.28	118±15.67	109.65±22.62	

NFC=Non-fiber carbohydrate. RDPR=RDP: RUP ratio. NFC1=NFC 30%; NFC2=NFC 35%; NFC3=NFC 40%; RDPR1=50:50 RDP: RUP ratio; RDPR2=55:45 RDP: RUP ratio; RDPR3=60:40 RDP: RUP ratio. VFA=Volatile fatty acids

Individual VFAs consisted of short-chain fatty acids (acetate, propionate, and n-butyrate), branched-chain fatty acids (iso-butyrate and iso-valerate), and n-valerate. The interaction between RDPR and NFC affected the molar proportion of acetate (p=0.018), but did not affect the molar proportion of propionate (p=0.668). The NFC2 and NFC3 combinations (35% NFC and 40% NFC, respectively) with RDPR treatment resulted in a high molar proportion of acetate. High NFC levels decreased the molar proportion of n-butyrate (p=0.040). There was an interaction between the two factors, both for the molar proportion of iso-butyrate (p=0.017) and n-valerate (p=0.031), but no significant difference in the molar proportion of iso-valerate (p=0.210). The acetate to propionate ratio increased with increasing NFC levels (p=0.020). Meanwhile, methane production was affected by both the RDPR and NFC treatments (p=0.010 and p=0.002). The molar proportion of VFA (mol/100 mol), acetate to propionate ratios, and methane production is detailed in Table-3.

**Table 3:** Molar proportion of VFA, acetate to propionate ratios, and methane production of experimental diet.

Parameters	RDPR	NFC			Average ± SD

		NFC1	NFC2	NFC3	
Acetate (%)	RDPR1	49.73^b^	57.34^a^	59.12^a^	53.94 ± 4.17
	RDPR2	54.21^ab^	56.57^a^	58.61^a^	57.88 ± 3.42
	RDRP3	57.87^a^	59.72^a^	57.99^a^	58.58 ± 1.85
	Average ± SD	55.40 ± 5.62	56.47 ± 2.39	58.53 ± 1.81	
Propionate (%)	RDPR1	20.25	18.03	18.50	18.93 ± 1.99
	RDPR2	18.71	18.09	18.39	18.40 ± 1.81
	RDRP3	18.43	17.87	18.20	18.17 ± 1.79
	Average ± SD	19.13 ± 1.79	18.00 ± 2.06	18.36 ± 1.62	
n-butyrate (%)	RDPR1	13.29	11.74	11.59	12.20 ± 1.34
	RDPR2	12.62	12.54	12.11	12.42 ± 0.74
	RDRP3	12.02	11.45	11.59	11.69 ± 0.79
	Average ± SD	12.64 ± 1.05^a^	11.91 ± 0.98^b^	11.76 ± 0.86^b^	
Iso-butyrate (%)	RDPR1	5.45^ab^	5.10^ab^	4.20^ab^	5.35 ± 1.41
	RDPR2	5.90^a^	5.89^a^	2.91^b^	4.85 ± 1.53
	RDRP3	4.70^ab^	3.56^ab^	4.71^ab^	3.94 ± 1.48
	Average ± SD	4.92 ± 1.35	4.90 ± 1.97	4.32 ± 1.30	
Iso-valerate (%)	RDPR1	5.69	4.79	4.56	5.01 ± 1.10
	RDPR2	5.17	4.95	5.44	5.19 ± 0.53
	RDRP3	4.31	5.10	5.18	4.87 ± 0.94
	Average ± SD	5.05 ± 0.99	4.95 ± 1.01	5.06 ± 0.67	
n-valerate (%)	RDPR1	5.59^a^	3.00^b^	2.03^b^	3.54 ± 1.95
	RDPR2	3.40^ab^	1.96^b^	2.54^b^	2.63 ± 1.05
	RDRP3	2.66^b^	2.29^b^	2.32^b^	2.42 ± 0.54
	Average ± SD	3.88 ± 1.57	2.42 ± 1.23	2.29 ± 0.54	
Acetate to propionate ratios	RDPR1	2.47	3.24	3.24	2.98 ± 0.61
	RDPR2	2.94	3.17	3.20	3.10 ± 0.38
	RDRP3	3.16	3.38	3.21	3.25 ± 0.37
	Average ± SD	2.86 ± 0.46^b^	3.26 ± 0.50^a^	3.22 ± 0.34^a^	
CH_4_ (mM)	RDPR1	7.73	5.41	6.54	6.56 ± 1.37^b^
	RDPR2	7.27	6.02	7.10	6.80 ± 1.25^b^
	RDRP3	9.18	6.96	7.92	8.02 ± 1.44^a^
	Average ± SD	8.06 ± 1.16^a^	6.13 ± 1.40^b^	7.19 ± 1.22^a^	

Means in the same row and column with different superscripts significantly different (p < 0.05). NFC = Non-fiber carbohydrate. RDPR = RDP: RUP ratio. NFC1 = NFC 30%; NFC2 = NFC 35%; NFC3 = NFC 40%; RDPR1 = 50:50 RDP: RUP ratio; RDPR2 = 55:45 RDP: RUP ratio; RDPR3 = 60:40 RDP: RUP ratio

### Rumen microbes and MPS

The rumen microbe population indicates how nutrient degradation occurs in the rumen. The protozoa, total bacteria population, and MPS results are presented in Table-4. Both main factors and their interaction did not significantly affect the protozoa nor the total bacteria population (p>0.05). MPS was significantly affected by both the NFC and RDPR treatments (p=0.049), but these two factors did not interact. NFC2 (35% NFC) produced higher MPS than NFC1 and NFC3. In contrast, RDPR3 (RDPR of 60:40) had higher MPS than RDPR1 and RDPR2.

**Table 4:** Total bacteria and protozoa population, microbial protein synthesis of experimental diet.

Parameters	RDPR	NFC			Average±SD

		NFC1	NFC2	NFC3	
Total bacteria population (log CFU/ml)	RDPR1	8.17	8.20	7.99	8.12±0.34
	RDPR2	7.97	8.34	8.12	8.14±0.25
	RDRP3	8.22	8.16	7.80	8.06±0.37
	Average±SD	8.12±0.30	8.23±0.24	7.97±0.37	
Protozoa population (log cell/ml)	RDPR1	6.51	6.62	6.57	6.57±0.11
	RDPR2	6.59	6.59	6.64	6.60±0.10
	RDRP3	6.55	6.57	6.65	6.59±0.12
	Average±SD	6.55±0.10	6.60±0.09	6.62±0.13	
MPS (mg/10 ml)	RDPR1	7.17	7.07	6.80	7.01±0.83^ab^
	RDPR2	6.46	7.40	6.96	6.94±0.90^b^
	RDRP3	7.23	8.21	7.09	7.51±1.12^a^
	Average±SD	6.95±0.96^b^	7.56±1.12^a^	6.95±0.70^b^	

Means in the same row or column with different superscripts are significantly different (p<0.05). NFC=Non-fiber carbohydrate. RDPR=RDP: RUP ratio. NFC1=NFC 30%; NFC2=NFC 35%; NFC3=NFC 40%; RDPR1=50:50 RDP: RUP ratio; RDPR2=55:45 RDP: RUP ratio; RDPR3=60:40 RDP: RUP ratio. CFU=Colony forming unit; MPS=Microbial protein synthesis

### Dry matter and organic matter digestibility (DMD and OMD)

DMD and OMD are presented in Table-5, neither of which were affected by the interaction between RDPR and NFC. RDPR did not significantly influence DMD (p=0.314) and OMD (p=0.359), but NFC levels (p=0.000 and p=0.001 for DMD and OMD respectively). NFC3 produced the highest DMD and OMD, but NFC1 and NFC2 showed similar results. The mean DMD and OMD ranged from 61.46 to 76.22% and 60.09 to 75.12%, respectively.

**Table 5:** Dry matter and organic matter digestibility of the experimental diet.

Parameters	RDPR	NFC			Average±SD

		NFC1	NFC2	NFC3	
DMD (%)	RDPR1	61.46	68.00	71.73	67.07±8.36
	RDPR2	65.44	67.06	71.29	67.93±7.29
	RDRP3	65.21	68.21	76.22	69.88±8.45
	Average±SD	64.04±8.00^b^	67.76±6.87^b^	73.08±6.53^a^	
OMD (%)	RDPR1	60.09	67.04	70.18	65.77±8.63
	RDPR2	64.25	65.63	69.59	66.49±7.58
	RDRP3	63.59	66.96	75.12	68.56±8.88
	Average±SD	62.64±8.41^b^	66.54±7.26^b^	71.63±6.91^a^	

Means in the same row with different superscripts significantly different (p<0.05). NFC=Non-fiber carbohydrate. RDPR=RDP: RUP ratio. NFC1=NFC 30%; NFC2=NFC 35%; NFC3=NFC 40%; RDPR1=50:50 RDP: RUP ratio; RDPR2=55:45 RDP: RUP ratio; RDPR3=60:40 RDP: RUP ratio. DMD=Dry matter digestibility, OMD=Organic matter digestibility

## Discussion

### Fermentation characteristics

Rumen pH describes the rumen condition; it is one of the factors that determine the fermentation process. Suitable pH conditions will support rumen microbial life, such that fermentation can proceed. This study showed that the rumen pH value was not affected by increases in RDPR and NFC; similar results were also reported by Pilachai *et al*. [32] and Wei *et al*. [18]. This insignificant effect of RDPR and NFC is due to the rumen microbes’ utilization of N and VFAs for synthesis, which does not acidify the rumen. The rumen pH values noted in this study were in the range of 6.92-6.97, indicating that there was no acidosis in the rumen, even at the highest RDPR and NFC levels. A pH of 6.0-7.0 is the ideal rumen pH to support the growth of rumen microbes and their activity in producing fermentation products such as NH_3_ and VFAs; under these conditions, the activity and composition of rumen microbes are not affected [29]. This pH range is also ideal for cellulolytic microbial activity [33,34].

Ammonia (NH_3_) is the main N source for MPS, and the NH_3_ concentration describes the amount of N used by microbes [35]. According to McDonald *et al*. [30], the optimal NH_3_ concentration ranges from 85 to 300 mg/l, or equivalent to 4.9 to 17.6 mM. The minimum ammonia concentration for microbial protein production is 3.6 mM [36]. In this study, the NH_3_ concentrations ranged from 7.60 to 8.11 mM, indicating that the protein ration was degraded to form sufficient NH_3_ for microbial growth. Part of the NH_3_ produced is absorbed, reused (N recycling), and plays an important role in the metabolic process of ruminants [37]. Our results demonstrated that the NH_3_ concentration was not affected by the RDPR and NFC treatments. A similar result was also reported by Savari *et al*. [12]; however, these results differ from those of the previous studies, which showed that the NH_3_ concentration tended to increase as RDPR increases [6,32]. The insignificant effect of RDPR on NH_3_ concentration may be caused by the C chain balance released from NFC fermentation used for microbial synthesis. The insignificant difference in NH_3_ concentration may also result from the small RDPR levels used in this study [12].

VFAs are the primary energy source for ruminants. The total VFA concentration was not different between the treatments. This result was similar to those of Savari *et al*. [12] and Hall *et al*. [6], who found that total VFA concentration was not affected by RDPR nor NFC levels. The total VFA concentration in this study ranged from 100.96 to 121.20 mM, which is within the normal range for concentrate-forage-based rations [31]. Several factors can affect total VFA concentration, including the rate of rumen fermentation, the amount of soluble substrate, the rate of feed consumption, VFA absorption, and liquid or solid passage [38]. Nonetheless, in this study, the amount of fermented NFC and RDP did not significantly affect the total VFA concentration; this was due to the fermentation products of NFC and RDP being released in a balanced proportion and used by microbes for protein synthesis. In the rumen, VFAs are absorbed by rumen epithelial cells. Ruminal VFA absorption is influenced by the VFA production and absorption balance in the rumen, epithelial permeability to VFAs, and the blood transport of VFAs from epithelial cells [39].

Molar proportions of VFAs are affected by substrate composition, substrate availability, rate of depolymerization, and availability of rumen microbial species [40]. The interaction between NFC (35% NFC and 40% NFC) with all levels of RDPR produced the highest molar proportion of acetate. Treatments NFC2 and NFC3 contained cassava meal at 20-30% DM. Cassava meal is rich in carbohydrates, especially pectin, producing a high proportion of acetate [41]. This finding aligns with previous studies, which reported a high molar proportion of acetate in pectin-rich rations [42,43].

Propionate is a substrate for gluconeogenesis, as the primary source of glucose in dairy cattle, whereas acetate and n-butyrate are sources of long-chain fatty acid synthesis [44]. In this study, the molar proportion of propionate was not affected by NFC nor RDPR. An insignificant effect of RDPR on the molar proportion of propionate has also been reported in several previous studies [35,45]. Conversely, Ma *et al*. [46] noted that the molar proportion of propionate increases as NFC increases; this different result is likely due to the use of different types of NFC. Nonetheless, other studies demonstrated that differences in the NFC content in rations play a small role in influencing rumen fermentation, especially propionate [47-49]. In addition, the molar proportion of n-butyrate decreased as the NFC level increased. A high level of NFC, rich in pectin content, is more fermentable than NFC rich in other forms of carbohydrates; this condition leads to higher acetate formation and lower formation of butyrate and lactate [50]. High molar proportions of butyrate are found in diets rich in saccharides, glucose, and disaccharides (fructose and sucrose) [6,20,51]. Butyrate plays a role in stimulating rumen papillae growth and proliferation [52].

Branched-chain VFAs (iso-butyrate, 2-methyl butyrate, and iso-valerate) are produced from oxidative deamination and decarboxylation of branched amino acids, such as valine, isoleucine, and leucine. Meanwhile, n-valerate is produced from carbohydrates and the carboxylic acid of proline, arginine, lysine, and methionine [53]. Iso-butyrate, iso-valerate, and n-valerate supply a C skeleton for the biosynthesis of branched-chain amino acids (valine, isoleucine, leucine, and proline) by cellulolytic bacteria [54]. This study found that the interaction between RDPR and NFC had a significant effect on the molar proportions of iso-butyrate and n-valerate; however, they did not affect iso-valerate. This result differed from a previ­ous study, which reported no effect of the interaction between NFC and RDPR on iso-butyrate, n-valer­ate, and iso-valerate [46]. Moreover, Pormalekshahi *et al*. [45] reported that the molar proportion of iso-valerate and n-valerate were not affected by different levels of RDPR. Nonetheless, replacing the RDP source from true protein with NPN (non-protein N) decreases iso-butyrate [55].

The interaction between NFC and RDPR on the molar proportion of iso-butyrate and n-valerate may be because the combination of these two factors can supply the amino acids required for the formation of iso-butyrate and n-valerate. Iso-butyrate and n-valerate are the main components required for the growth of cellulolytic bacteria, especially *Bacteroides succinogenes*. Meanwhile, *Ruminococcus flavefaciens* requires iso-butyrate and iso-valerate, and *R. albus* requires iso-butyrate and 2-methyl butyrate [56]. The proportion of n-valerate was low in rations with high NFC levels (NFC2 and NFC3) for all RDPR levels; this may be caused by the treatment combinations’ high pectin and low sugar contents. According to Li *et al*. [57], high molar n-valerate is produced from rations containing high concentrates and sugar.

The acetate to propionate ratio increases with increasing NFC levels. The high availability of readily fermentable carbohydrates increases the amount of cellulolytic bacteria, DM digestibility, and the acetate to propionate ratio [2]. Pectin is a component of the neutral detergent soluble fiber, and its fermentation results in a high acetate to propionate ratio and lower lactate levels, although it does not lower rumen pH [4]. A high availability of branched-chain VFAs (iso-butyrate, iso-valerate, and n-valerate) is required to grow cellulolytic bacteria [56]. Increasing cellulolytic bacteria activity increases acetate production, leading to increases of the acetate to propionate ratio [58,59]. The acetate propionate ratio is positively correlated with methane emissions (CH_4_) from the fermentation process in the rumen and is used as an indicator of CH_4_ production [60]. The synthesis of acetate and butyrate produces hydrogen, whereas propionate synthesis uses hydrogen. Increasing the acetate and butyrate rumen concentration increases the hydrogen available for methanogenic bacteria to synthesize CH_4_ [61]. Estimations of CH_4_ based on VFA molar proportions influence the RDPR and NFC treatments, leading to high CH_4_ estimates in the NFC2, NFC3, and RDPR3 treatments.

### Rumen microbes and MPS

The rumen microbes consist of bacteria, protozoa, and fungi. The rumen microbial population is influenced by the rumen pH, temperature, buffer capacity, osmotic pressure, DM content, and oxidation-reduction potential [62]. Neither RDPR nor NFC affected the total bacteria and protozoa populations. The total bacteria population was 7.97-8.22 log CFU/mL, lower than that recommended by McDonald *et al*. [30] for a normal rumen bacteria population in ruminants (9-10 log CFU/mL). The lower total bacteria population in this study is likely due to the rations’ low crude fiber content, required to achieve the high NFC levels in the ration formulation. Low bacteria populations (8.78-8.41 log CFU/mL) associated with high NFC treatments consisting of corn and DDGS concentrate have also been previously reported [63]. The mean protozoa population in this study was 6.55-6.62 log cells/mL, within the normal range (5-6 log cells/mL) according to McDonald *et al*. [30]. An insignificant effect of carbohydrate type on the rumen protozoa population has also been previously reported [64]. In contrast, Vakili *et al*. [65] reported that NFC supplementation decreases the methanogenic bacteria and protozoa populations. Furthermore, they reported that the type of starch can reduce methanogenesis in the rumen.

Protozoa and rumen bacteria have different feeding behaviors with respect to protein digestion. Rumen bacteria are essential in degrading soluble protein, whereas protozoa degrade insoluble protein. In addition, as a predator, protozoa digest bacteria and fungi, and have a limited ability to assimilate peptides and amino acids [65]. Protozoa release more amino acids and peptides than bacteria in the rumen, but they cannot synthesize amino acids from ammonia [11]. According to Ferrand-Theix [66], protozoa defaunation increases N utilization by increasing the supply of amino acids to the small intestine and decreasing urinary N excretion into the environment. Factors that affect rumen protozoa include feed composition, rumen pH, and feeding frequency [67].

Microbial protein is the primary protein source for ruminants. Approximately 80-90% of amino acid requirements are supplied by microbial protein with 80% digestibility [11,68]. MPS is influenced by the availability of energy from fermented carbohydrates in the rumen and N in the form of NH_3_ [11]. Both RDPR and NFC significantly affected MPS, but there was no interaction between the two factors. Treatment RDPR3 (RDPR of 60:40) produced higher MPS than RDPR1 and RDPR2, whereas treatment NFC2 (35% NFC) produced higher MPS in comparison to NFC1 and NFC3. A previous study reported that an increase in RDP and rumen degradable starch increased recycled N, thereby increasing the supply of MPS to the small intestine and improving microbial protein efficiency [69]. High NFC in the rations may increase MPS since it provides quickly fermentable carbohydrates and energy [7].

In this study, MPS was 6.94-7.56 mg/10 mL; this was lower than the range reported by Putri *et al*. [70], who reported a MPS of 10 mg/10 mL produced from a legume-based ration with 16% protein content and different RDPR levels (55:45, 60:40, and 65:35). This difference in MPS is due to the difference in feed ingredients (leguminous vs. concentrate) and types of NFC used in the rations. Although the amino acids and NPN in the rations were not analyzed, we suggest that both components have a role in improving MPS. Changing RDP from true protein to NPN reduces MPS in lactating dairy cows [55], and in *in vitro* studies, feeding RDP as free amino acids and peptides promote microbial growth and efficiency [71]. The type of NFC also affects MPS. The high pectin and sugar types of NFC depressed MPS more than the starch type of NFC. The N efficiency for MPS varies, influenced by fermented carbohydrates’ energy efficiency [72]. An average of 20 g of bacterial protein is synthesized per 100 g of organic matter fermented in the rumen [10]. The primary nutrients needed for MPS are carbohydrates and proteins. The balance of energy and N supply in MPS can be calculated by a synchronization index, which is calculated based on the organic matter and N degraded per hour [73]. A high synchronization index increases MPS [74]. In contrast, the low availability of essential micro minerals and vitamins become limiting factors for MPS production [2]. Therefore, supplementation of sulfur increases MPS efficiency [75].

### DMD and OMD

The DMD and OMD values ranged from 61.46 to 76.22% and 60.09 to 75.12%, respectively, indicating that the rations were moderately digestible. NFC provides easily digestible carbohydrates, including starch, simple sugars, beta-glucan, galactan, and pectin. Easily digestible carbohydrates provide energy and increase N utilization to synthesize microbial protein [76]. Rations with a higher energy supply produce higher DMD and OMD [7]. In this study, the highest DMD and OMD were noted with the NFC3 treatment (40% NFC), which used 30% of cassava in the ration as a source of NFC. The cassava cell wall is dominated by pectin substances, such as arabinans and xylogalacturonans [42]. According to Zhao *et al*. [8], DMD and OMD are higher under pectin treatments than starch and disaccharide-diet carbohydrates due to the rate of passage in the digestive tract [20]. In this study, the RDPR treatment did not affect DMD and OMD. The different degradability in the rumen was compensated by different post-ruminal digestibility, which resulted in similar total DMD and OMD. One previous study reported that an increasing RDPR level improves DMD and OMD [70]; this is true for rations high in fiber that rely mainly on rumen fiber degradation, but this was not the case in the current study.

## Conclusion

Tropical dairy rations formulated using a combination RDPR of 60:40 with 35% NFC resulted in the best synchronization of available protein and energy for MPS and digestion activity in the rumen. Increasing NFC levels up to 40% resulted in higher DMD and OMD, but lower MPS. High NFC levels increased the acetate to propionate ratio suitable for milk fat synthesis. Further *in vivo* studies are needed to determine the effect of 60:40 RDPR and 35% NFC on milk production and quality and the use of different NFC types in dairy rations, such as sugar, starch, and pectin.

## Authors’ Contributions

IGP and DD: Conceptualization, formulated the experimental design, revised and finalized the manuscript. AR: Experimental work at the laboratory, data analysis, and drafted the manuscript. All authors read and approved the final manuscript.
